# Supplementary material for: Seminoma and Embryonal Carcinoma Footprints Identified by Analysis of Integrated Genome-Wide Epigenetic and Expression Profiles of Germ Cell Cancer Cell Lines
Source: PLoS One. 2014 Jun 2;9(6):e98330. doi: 10.1371/journal.pone.0098330 (PMC4041891; doi:10.1371/journal.pone.0098330)
Supplement: File S1 — ZIP file containing DMRforPairs output for significant regions. Please start from the html files. (ZIP) [file pone.0098330.s008.zip › figures/36868.pdf]

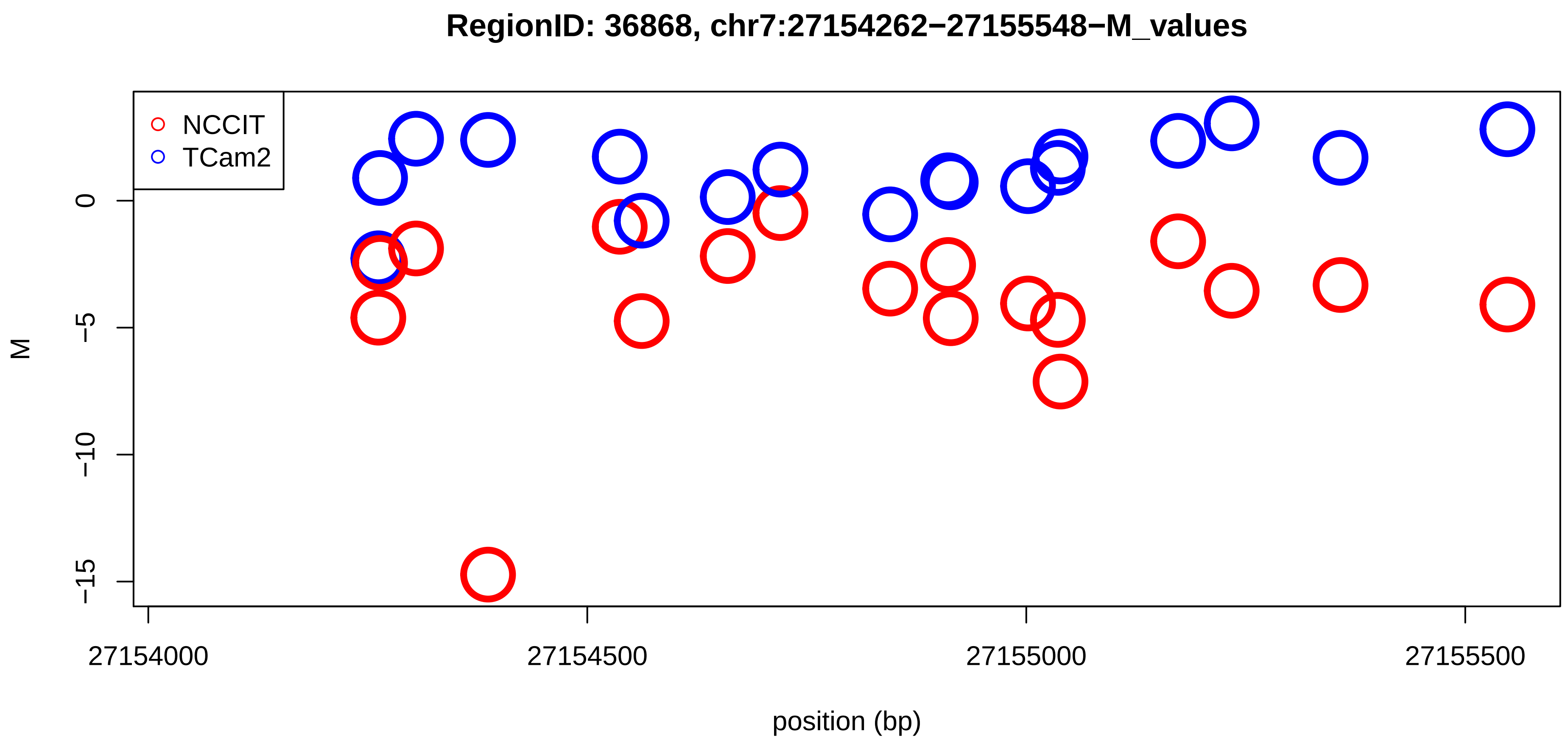
RegionID: 36868, chr7:27154262−27155548−M_values
NCCIT
TCam2
M
0
−5
−10
−15
27154000
27154500
27155000
27155500
position (bp)

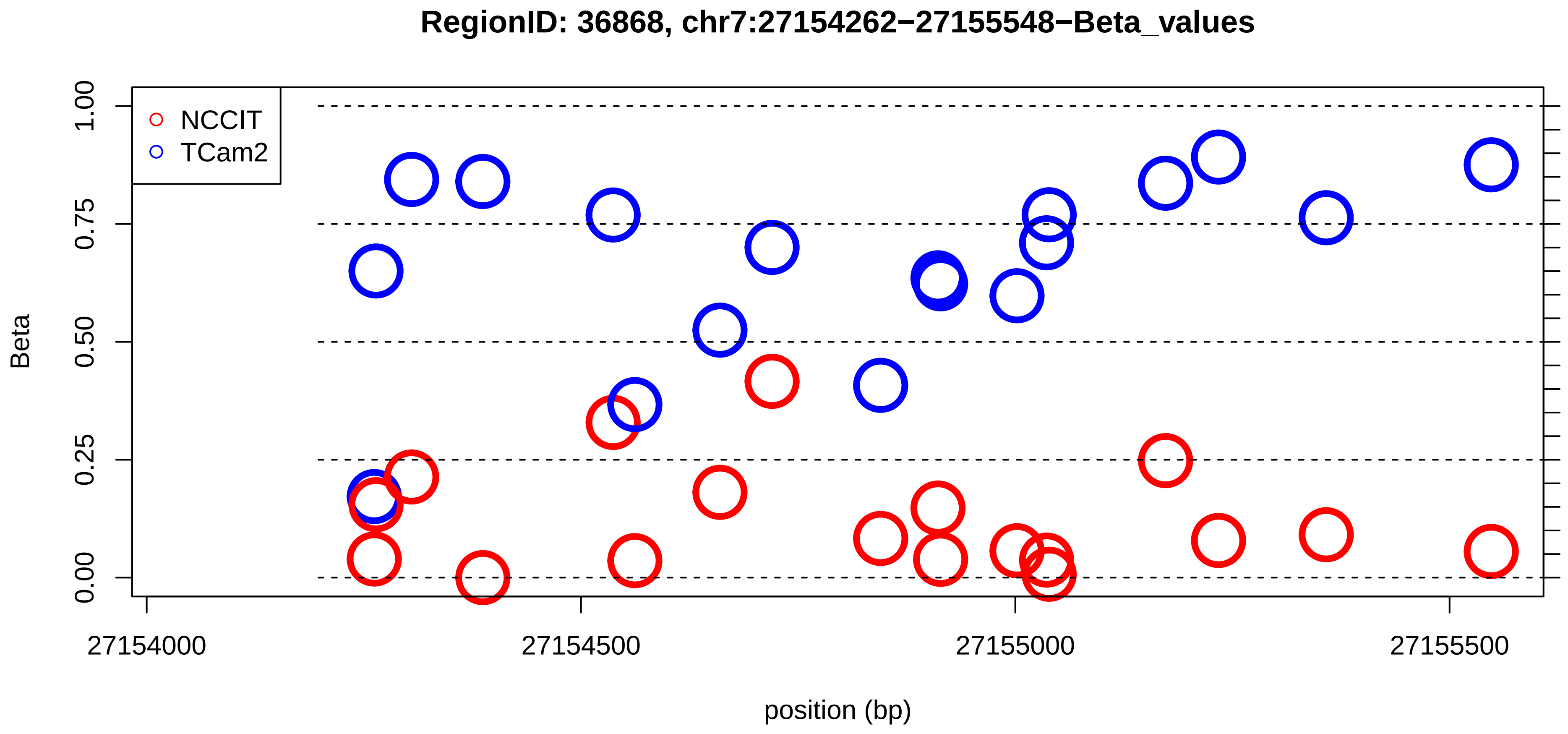
RegionID: 36868, chr7:27154262−27155548−Beta_values
NCCIT
TCam2
Beta
0.00
0.25
0.50
0.75
1.00
27154000
27154500
27155000
27155500
position (bp)

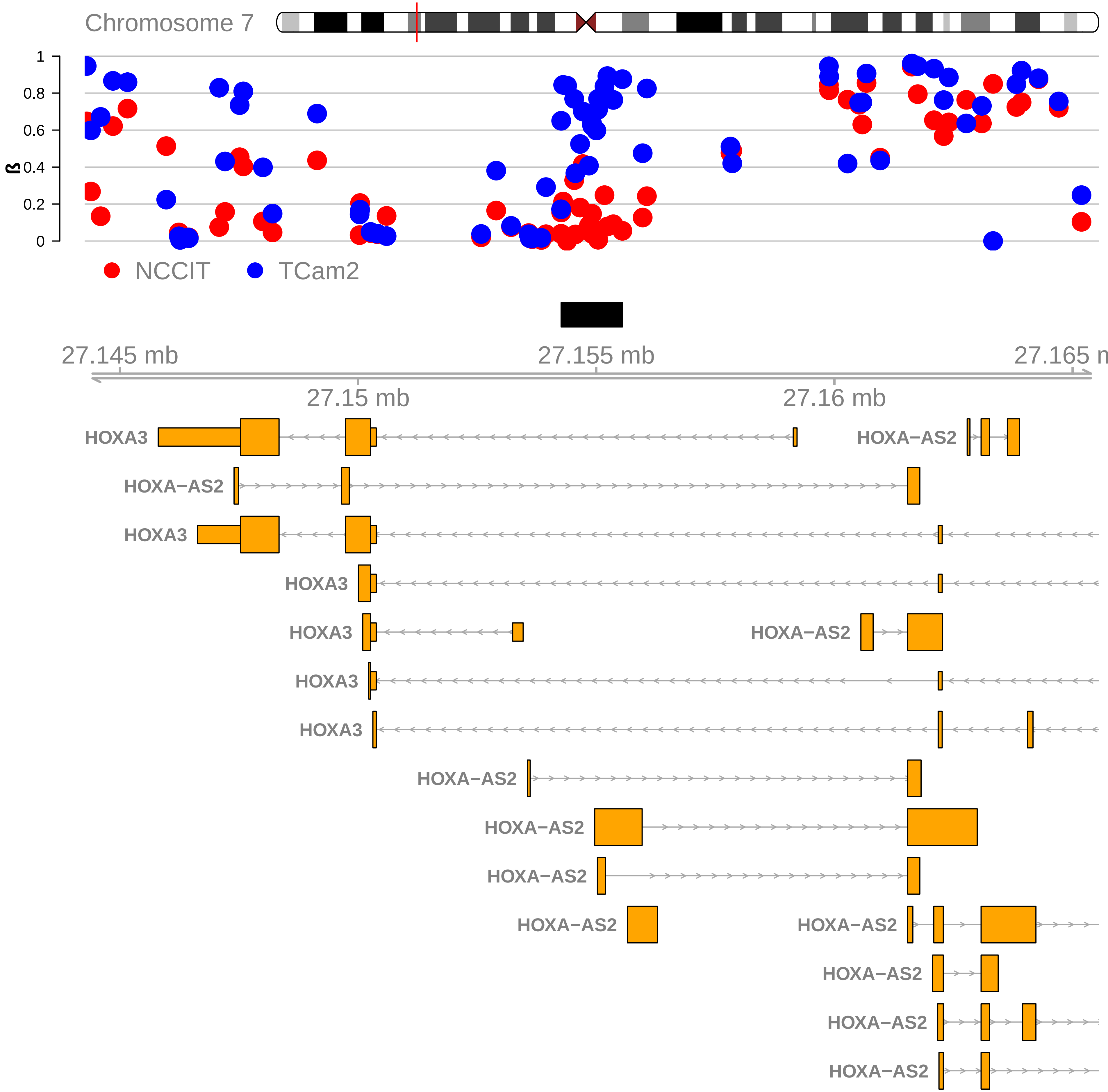

Chromosome 7
1
0.8
0.6
0.4
0.2
0
ß
NCCIT
TCam2
27.145 mb
27.155 mb
27.165 mb
27.15 mb
27.16 mb
HOXA3
HOXA−AS2
HOXA−AS2
HOXA3
HOXA3
HOXA3
HOXA−AS2
HOXA3
HOXA3
HOXA−AS2
HOXA−AS2
HOXA−AS2
HOXA−AS2
HOXA−AS2
HOXA−AS2
HOXA−AS2
HOXA−AS2
